# Detection of SARS-CoV-2 infection by saliva and nasopharyngeal sampling in frontline healthcare workers: An observational cohort study

**DOI:** 10.1371/journal.pone.0280908

**Published:** 2023-01-27

**Authors:** Naomi F. Walker, Rachel L. Byrne, Ashleigh Howard, Elissavet Nikolaou, Madlen Farrar, Sharon Glynn, Katerina S. Cheliotis, Ana I. Cubas Atienzar, Kelly Davies, Jesús Reiné, Zalina Rashid-Gardner, Esther L. German, Carla Solórzano, Tess Blandamer, Lisa Hitchins, Christopher Myerscough, Bradford D. Gessner, Elizabeth Begier, Andrea M. Collins, Mike Beadsworth, Stacy Todd, Helen Hill, Catherine F. Houlihan, Eleni Nastouli, Emily R. Adams, Elena Mitsi, Daniela M. Ferreira, on behalf of the SAFER investigators

**Affiliations:** 1 Clinical Sciences, Liverpool School of Tropical Medicine, Liverpool, United Kingdom; 2 Tropical and Infectious Diseases Unit, Liverpool University Hospitals NHS Foundation Trust, Liverpool, United Kingdom; 3 Centre for Drugs and Diagnostics, Tropical Disease Biology, Liverpool School of Tropical Medicine, Liverpool, United Kingdom; 4 Department of Infection and Immunity, Murdoch Children’s Research Institute, Parkville, Victoria, Australia; 5 Oxford Vaccine Group, University of Oxford, Oxford, United Kingdom; 6 NIHR Liverpool and Broadgreen Clinical Research Facility, Liverpool University Hospitals NHS Foundation Trust, Liverpool, United Kingdom; 7 Pfizer Vaccines, Collegeville, Pennsylvania, United States of America; 8 National Institute for Health Research North West Coast, Liverpool, United Kingdom; 9 Liverpool University Hospitals NHS Foundation Trust, Liverpool, United Kingdom; 10 Liverpool Health Partners, Liverpool, United Kingdom; 11 Department of Clinical Virology, University College London Hospitals, London, United Kingdom; 12 Department of Infection, Immunity and inflammation, Great Ormond Street Institute of Child Health, University College London, London, United Kingdom; Waseda University: Waseda Daigaku, JAPAN

## Abstract

**Background:**

The SARS-CoV-2 pandemic has caused an unprecedented strain on healthcare systems worldwide, including the United Kingdom National Health Service (NHS). We conducted an observational cohort study of SARS-CoV-2 infection in frontline healthcare workers (HCW) working in an acute NHS Trust during the first wave of the pandemic, to answer emerging questions surrounding SARS-CoV-2 infection, diagnosis, transmission and control.

**Methods:**

Using self-collected weekly saliva and twice weekly combined oropharyngeal/nasopharyngeal (OP/NP) samples, in addition to self-assessed symptom profiles and isolation behaviours, we retrospectively compared SARS-CoV-2 detection by RT-qPCR of saliva and OP/NP samples. We report the association with contemporaneous symptoms and isolation behaviour.

**Results:**

Over a 12-week period from 30^th^ March 2020, 40·0% (n = 34/85, 95% confidence interval 31·3–51·8%) HCW had evidence of SARS-CoV-2 infection by surveillance OP/NP swab and/or saliva sample. Symptoms were reported by 47·1% (n = 40) and self-isolation by 25·9% (n = 22) participants. Only 44.1% (n = 15/34) participants with SARS-CoV-2 infection reported any symptoms within 14 days of a positive result and only 29·4% (n = 10/34) reported self-isolation periods. Overall agreement between paired saliva and OP/NP swabs was 93·4% (n = 211/226 pairs) but rates of positive concordance were low. In paired samples with at least one positive result, 35·0% (n = 7/20) were positive exclusively by OP/NP swab, 40·0% (n = 8/20) exclusively by saliva and in only 25·0% (n = 5/20) were the OP/NP and saliva result both positive.

**Conclusions:**

HCW are a potential source of SARS-CoV-2 transmission in hospitals and symptom screening will identify the minority of infections. Without routine asymptomatic SARS-CoV-2 screening, it is likely that HCW with SARS-CoV-2 infection would continue to attend work. Saliva, in addition to OP/NP swab testing, facilitated ascertainment of symptomatic and asymptomatic SARS-CoV-2 infections. Combined saliva and OP/NP swab sampling would improve detection of SARS-CoV-2 for surveillance and is recommended for a high sensitivity strategy.

## Introduction

The Severe Acute Respiratory Syndrome Coronavirus 2 (SARS-CoV-2), the causative agent of the coronavirus disease 2019 (COVID-19) pandemic, has caused an unprecedented strain on health systems worldwide, including in the United Kingdom (UK) National Health Service (NHS) and the frontline healthcare workers (HCW) it employs. HCW are particularly vulnerable to SARS-CoV-2 infection due to their frequent close proximity to infectious COVID-19 patients [1]. The risk of transmission of SARS-CoV-2 from infected HCW to their patients and colleagues whilst attending work was also recognized as a major threat to infection prevention and control. Transmission-based precautions (including personal protective equipment) were implemented widely, for the protection of the staff and their patients.

Whilst reverse transcription polymerase chain reaction (RT-qPCR) assays for SARS-CoV-2 on respiratory samples have proven the mainstay of clinical diagnosis, in the early stages of the pandemic testing capacity was extremely limited and was reserved only for hospitalised individuals meeting the COVID-19 case definition. The UK COVID-19 case definition (Box 1) evolved during the outbreak, losing the initial geographic restrictions on 12^th^ March 2020 as transmission became widespread. With coordinated national and global efforts, diagnostic testing capacity was scaled up, substantially. Regular asymptomatic screening of HCW became standard practice in the NHS, utilising either respiratory nose/throat swabs or saliva samples, however, there has not been consensus on the optimal frequency or sample type for surveillance.

**Box 1. UK COVID-19 Case Definitions and guidance**

**Stay at home guidance (for HCW & public)**

issued by Chief Medical Officer, 12^th^ March 2020:

    a. A new continuous cough OR

    b. High temperature (of 37·8 degrees centigrade or higher)

regardless of travel history or contact with confirmed cases.*

**UK government testing advice**

(from https://www.gov.uk/coronavirus, accessed 12^th^ January 2021):

**If you have any coronavirus symptoms:**

- a high temperature
- a new, continuous cough
- a loss of, or change to, your sense of smell or taste**

**Get a test** **and stay at home**

    NB: Guidance was initially to stay at home for a minimum of 7 days as no community or occupational testing was available to support this.

                        *Geographic restrictions were removed on 12^th^ March 2020

    **    This additional symptom criteria was introduced from 18^th^ May 2020

SARS-CoV-2 Acquisition in Frontline Health Care Workers–Evaluation to Inform Response (SAFER) was a cohort study designed to prospectively collect data on infections in HCW, in order to inform control strategies in healthcare settings. In this retrospective analysis of a subgroup of SAFER Liverpool participants from March–July 2020, before routine HCW surveillance was implemented, we evaluated the prevalence of SARS-CoV-2 RNA detected by weekly saliva or twice weekly combined oropharyngeal/nasopharyngeal (OP/NP) self-testing by RT-qPCR, the occurrence of symptoms of infection and self-isolation. Laboratory analysis was not conducted in real time and HCW followed national guidance for isolation based on symptoms. We and others have previously demonstrated the utility of saliva as a reliable alternative to nose/throat swabbing for the detection of SARS-CoV-2 in symptomatic individuals [2–5]. Here, we compare SARS-CoV-2 detection by RT-qPCR in saliva and OP/NP swab samples collected routinely in a cohort of HCW and report the association with symptom profiles. We also describe the association between symptoms, SARS-CoV-2 infection and self-isolation behaviour in HCW at a time when routine asymptomatic SARS CoV-2 testing was not available.

## Materials and methods

### Ethics

Ethical approval was obtained from the NHS Health Research Authority South Central–Berkshire Research Ethics Committee (ref 20/SC/0147). All participants provided written informed consent.

### Participant recruitment and involvement

Participants were HCW in a variety of roles who enrolled onto the SAFER Study between 30^th^ March and 9^th^ April 2020 at Royal Liverpool University Hospital (RLUH), Liverpool University Hospitals NHS Foundation Trust. Eligible individuals were at least 18 years old, were working in a patient facing role for at least five hours for at least one day during the study period (12 weeks from enrolment) and did not have COVID-19 symptoms at the time of enrolment. Participants were invited from areas caring for COVID-19 patients (Accident and Emergency, the acute medical unit, infectious diseases and respiratory wards), and areas which aimed to be COVID-19 free (haematology, surgical wards). A target sample size (n = 100) was pragmatic, limited by the capacity of the research team to recruit the cohort over a relatively short time frame in an epidemic setting and the first 85 participants were invited to submit saliva in addition to OP/NP samples. Participants engaged with the study team through regular telephone and email communication. Participants were free to withdraw from the study at any point if they chose to do so, including if they were transferred or moved away from the study site. On withdrawal, participants could choose to either allow their existing data to remain in the study or be excluded. Inclusion in this subgroup analysis required the participant to have provided saliva samples.

### Sample collection and storage

Participants were asked to provide a self-collected OP/NP sample by swabbing the throat and then nasopharynx twice weekly when attending work, following instructions (see S1 Appendix). Each participant was observed performing collection using the correct technique at study enrolment prior to unsupervised sample collection at later timepoints. Swabs were transported at room temperature in 1ml of liquid amies (MWE, UK) to designated collection points within the hospital by participants on the day of collection and then transported to the laboratory daily by the research team, where they were stored at -80°C, until further analysis. Saliva (at least 500μl) was collected once per week into a sterile tube (SARSTEDT, USA) by passive drool using a plastic funnel at home. Participants were asked to store saliva samples in their home freezers (approximately -20°C) immediately, before transportation in cooler packs (provided by the research team) to the Liverpool School of Tropical Medicine (LSTM) laboratories for processing at monthly intervals, after which time they were also stored at -80°C, until analysis. Participants were instructed not to collect OP/NP samples during periods of self-isolation, however, they continued to collect weekly saliva samples during this time. Laboratory testing for SARS-CoV-2 infection was retrospective. OP/NP results were made available to participants after study follow up was complete.

### RNA extraction

Viral RNA from OP/NP swabs was extracted using the high-throughput Quick DNA/RNA™ viral MagBead kit (Zymo, USA), whereas viral RNA from saliva was extracted using the QIAamp Viral RNA Mini Kit (Qiagen, Germany), following in-house comparison. Both followed manufacturer’s instructions and an internal extraction control incorporated at the lysis stage (Genesig, UK). Once extracted, RNA samples were taken immediately for downstream application and stored on ice during RT-qPCR setup.

### SARS-CoV-2 RT-qPCR

For SARS-CoV-2 RT-qPCR detection, 8μl of extracted RNA was tested using the Genesig® Real-Time Coronavirus COVID-19 PCR assay (Genesig, UK) in a QuantStudio 5 thermocycler (ThermoFisher, USA). Samples were classified as RT-qPCR positive if both the internal extraction and the SARS-CoV-2 probes were detected at cycle threshold (Ct) <41. Genome copy numbers per ml (gcn/ml) were quantified using the manufacturer’s positive control (1·67 x 10^5^ gcn/μl) as a reference for threshold. Samples with invalid RT-qPCR internal extraction amplification results were re-extracted and re-run.

### Analytical sensitivity

The analytical sensitivity of saliva compared to OP/NP samples was compared by spiking the samples with serial dilutions of SARS-CoV-2 culture supernatant. The isolate REMRQ0001/human/2020/Liverpool propagated in VERO E6 cells and maintained, as previously described, was used for the serial dilutions [6]. Fourteen OP/NP swabs were stored in 1ml of amies preservation medium (Copan, Italy) and 4ml of saliva were self-collected from a confirmed RT-qPCR SARS-CoV-2 negative volunteer. A serial dilution series of SARS-CoV-2 ranging from 10^6^ to 10^−6^ plaque forming units per ml (pfu/ml) was used to spike 140μl of saliva and swab samples. The limit of detection (LOD) was determined by the lowest concentration for which all three RT-qPCR replicates amplified. For quantification of the gcn/ml, viral RNA of the serial dilutions was extracted using QIAmp Viral RNA mini kit (Qiagen, Germany) and the gcn/ml were calculated using the COVID-19 Genesig RT-qPCR kit (PrimerDesign, UK) with a ten-fold serial dilution of quantified specific in vitro-transcribed RNA (Genesig, UK).

### Symptom reporting, isolation and diagnostic tests

At the time of the study, SARS-CoV-2 testing of staff who did not meet the UK COVID-19 case definition (Box 1) was not routine, nor feasible due to lack of laboratory capacity and testing reagents. Participants were advised that if they had symptoms that met the COVID-19 case definition they should self-isolate and seek COVID-19 diagnostic testing via the staff testing service, when it became available (2^nd^ April 2020). Symptomatic participants continued study procedures, except for self OP/NP sample collection during periods of self-isolation. Symptom reporting was via a short questionnaire completed twice weekly, accompanying each sampling episode; participants could select from COVID-19 case definition symptoms and other respiratory illness symptoms, or enter other symptoms as free text. Periods of self-isolation and standard-of-care test results were reported by participants to the research team by telephone and/or email and recorded as they occurred. At monthly visits, information on symptoms, self-isolation periods, testing and hospitalisation were also reviewed. Symptoms associated with each sample collected were scored on a scale from 0 (no symptoms) to 6 (multiple symptoms consistent with the COVID-19 case definition) for the purposes of analysis (see Box 2). Participants were assigned the highest code for which they qualified.

**Box 2. Symptom score assigned for symptom status**

**Table:** 

**0**	Asymptomatic
**1**	Symptom (s), likely unrelated e.g. ankle pain
**2**	Symptom (s), non-specific e.g. Fatigue / headache / muscle aches
**3**	One respiratory symptom, not COVID case definition e.g. Cough (other), sore throat, coryza
**4**	More than one symptom including a respiratory symptom (not COVID-19 case definition symptom)
**5**	COVID-19 case definition symptom: New/continuous cough or fever or loss of taste/smell
**6**	More than one COVID-19 case definition symptom: new/continuous cough, fever, loss of taste or smell

### Statistical analysis

Analysis was in R (version 4.0.4) and Prism 8 (Graphpad). Two-tailed statistical tests were used to compare variables throughout the study. Non-parametric Mann-Whitney U test was used to compare two groups, unless otherwise stated. Correlations were assessed using Spearman’s correlation using raw, non log-transformed values.

## Results

### Participants and follow up

In total, 99 participants were enrolled into the SAFER Liverpool cohort. Of these, 85 participants provided both saliva and OP/NP samples over eight and twelve weeks respectively and are therefore included in this analysis. Participant characteristics are reported in Table 1. The majority (77·6%, n = 66) were female. Participants comprised doctors, nurses, healthcare assistants and allied health professionals (physiotherapists and occupational therapists). Retention in follow up was 84·7%, (n = 72) participants at four weeks, 81·2% (n = 69) participants at eight weeks and 74·1% (n = 63) (74·1%) participants at twelve weeks. A median of 21 OP/NP samples (interquartile range (IQR) 10·5–24·0) and 8 saliva samples (IQR 4·50–8·00) of a target of 24 and 8 respectively, were submitted per participant.

**Table 1:** Participant characteristics.

Total included participants (n)	85
Female, n (%)	66 (77·6)
Age, median years (IQR)	35.0 (27·5–46·5)
Role, n (%)	
Doctor	35 (41·2)
Nurse	32 (37·6)
Health Care Assistants	9 (10·6)
Allied health professional	6 (7·06)
Other	3 (3·53)
Experienced symptoms*, n (%)	
Any	40 (47·1)
COVID-19 case definition	9 (10·6)
Isolated during study period, n (%)	22 (25·8)
COVID-19 diagnosis during study**	3
COVID-19 hospitalisation	0

*Over 12 week study period.

**external to the study.

### COVID-19 diagnosis, symptoms and isolation status

**Fig 1:**
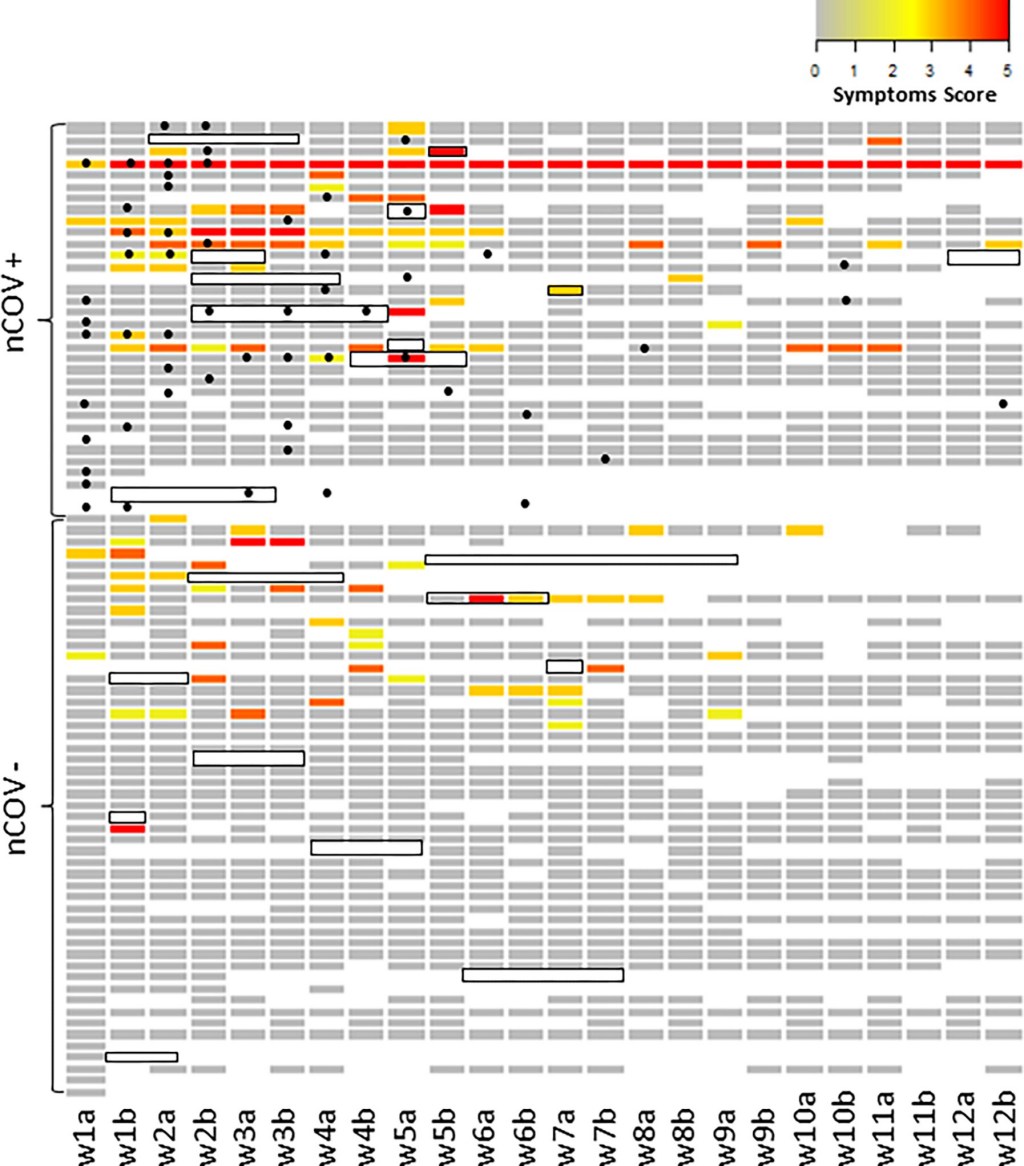
Symptoms, isolation and SARS-CoV-2 RNA status in HCW (n = 85) over a period of 12 weeks. Heatmap for recorded symptoms using a colour scale to represent symptoms score (see Box 2) from grey (0: Asymptomatic) to red (5: A symptom consistent with the COVID-19 case definition in red). No participants scored 6. The participant with symptoms recorded at every timepoint experienced loss of smell which persisted throughout the study period. Isolation period is indicated by a black box delineating timepoints (week 1–12). Participants were clustered into two groups, nCoV+ (positive for SARS-CoV-2 RNA by either diagnostic method), or nCoV- (SARS-CoV-2 RNA negative on results available). Timing of positive results are indicated by a black dot. First and second samples per week are labelled “a” and “b” respectively. Time points with missing data are shown in white.

**Fig 2:**
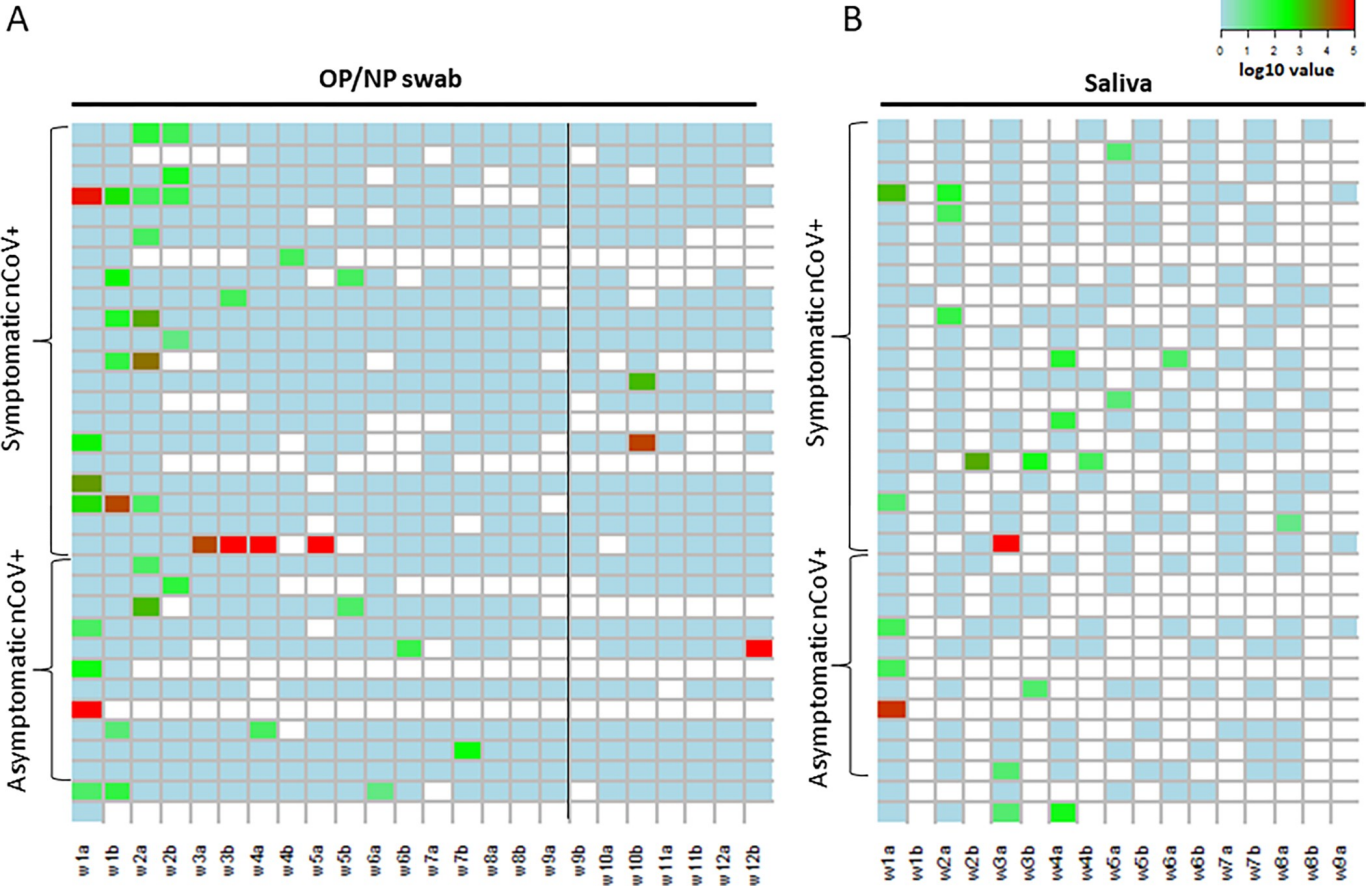
SARS-CoV-2 RNA detection by saliva and combined oropharyngeal/nasopharyngeal sample during study period. Timing of SARS-CoV-2 positivity by combined oropharyngeal/nasopharyngeal (OP/NP) and saliva samples over a period of 12- and 8-weeks, respectively, in SARS-COV-2 RNA positive (nCoV+) participants, clustered by symptom status across duration of study (symptomatic or asymptomatic). First and second samples per week are labelled “a” and “b”, respectively. **A)** Heatmap of log10 viral load value per OP/NP swab sample over surveillance period of 12 weeks (n = 25 nCoV+ by OP/NP swab: 15 symptomatic and 10 asymptomatic). **B)** Heatmap of log10 viral load value per saliva sample over the sampling period of 8 weeks (n = 17 nCoV+: 11 symptomatic and 6 asymptomatic). For two participants, insufficient symptom information was available to categorise as symptomatic or asymptomatic.

Of the 85 participants, 40·0% (n = 34, confidence interval (CI) 95% 31·3–51·8%) were positive for SARS-CoV-2 RNA on at least one study sample during the study period (Figs 1 and 2). Symptoms of any kind were reported by 47·1% (n = 40) participants during study follow up whilst attending work. However, only 44.1% (n = 15/34) participants with SARS-CoV-2 RNA detected on SAFER study samples reported symptoms within a 14-day period either side of their positive result (Table 2). Considering that symptoms may have been experienced prior to study enrolment, we excluded data for four asymptomatic participants who tested positive exclusively in the first week of the study, finding the proportion of participants with any symptoms associated with a positive SARS-CoV-2 results to be 50.0% (15/30). In participants with evidence of SARS-CoV-2 infection attending work, symptoms were predominantly respiratory (coryza, sore throat, cough) but most often not those included in the COVID-19 case definition.

**Table 2:** Symptom reporting in SARS-CoV-2 RNA positive participants during study follow up. (Excluding participants who were self-isolating for COVID-19 case definition symptoms).

Participants with positive results (OP/NP or saliva sample), n	34
Participants with symptoms associated with positive result*, n (% positive participants)	15 (44·1)
Fever	0 (0)
New continuous cough	1 (2.94)
Cough, other	5 (14·7)
Rhinorrhoea	9 (26·5)
Muscle aches	2 (5·88)
Joint Pain	2 (5·88)
Sore Throat	4 (11·8)
Headache	5 (14·7)
Chest Pain	0
Breathing Difficulty	0
Change in Taste or Smell**	5 (14·7)

*within 14 days of positive result.

**at any time during study follow up.

The timing of SARS-CoV-2 RNA detection and relationship with symptoms and self-isolation are shown in Fig 1. Most positive results were detected in the early weeks of the study period, which commenced shortly after the first national lockdown was declared (23^rd^ March 2020) and prior to universal use of face masks/coverings at all times for staff in hospital (introduced on 15^th^ June 2020). Nine participants reported symptoms consistent with the COVID-19 case definition and 14 participants reported COVID-19 testing during the 12-week period, of which only three participants received a confirmed diagnosis of COVID-19 by RT-qPCR of a nose/throat sample, for clinical purposes outside of the study setting. Periods of self-isolation were prevalent, reported by 25.9% (n = 22/85) participants. In seven incidences, self-isolation was reportedly due to a contact rather than due to symptoms. Only 29.4% (n = 10/34) participants who were SARS-CoV-2 RNA positive reported self-isolation. The median duration of isolation per participant was 6·50 (IQR 5·00–14·0) days.

### Analytical sensitivity

The analytical sensitivity in spiked samples indicated the LOD for saliva was 10^−2^ pfu/ml (≈ 2·0 x 10^1^ gcn/ml) and for the OP/NP swabs 10° pfu/ml (≈ 2·0 x 10^3^ gcn/ml). However, amplification of one or more replicates was recorded for saliva and OP/NP swabs to concentrations 10^−6^ (pfu/ml) and 10^−4^ (pfu/ml), respectively.

### Comparison of saliva and OP/NP samples for detection of SARS-CoV-2 infection by qPCR

SARS-CoV-2 RNA was detected in 3·02% (n = 43/1425) OP/NP samples and 4·15% (n = 22/530) saliva samples (Fig 2). A total of 17 participants were positive by only one sample at a single timepoint (Fig 2). Overall, eight participants were positive for SARS-CoV-2 RNA on both OP/NP and saliva sample, 17 participants were positive by OP/NP swab only and nine participants by saliva only. The median viral load in SARS-CoV-2 RNA positive participants with symptoms was 139 gcn/ml (IQR 31–3332 gcn/ml) and without symptoms was 64·4 gcn/ml (IQR 25·1–494·5 gcn/ml), p = 0·65. No correlation was found between viral loads and assigned symptom score (r = -0·12, p = 0·52). Ct values and corresponding viral loads are reported in the Supplementary Information (S1 and S2 Tables).

### Agreement of saliva and OP/NP swabs for SARS-CoV-2 detection in a paired sample cohort

**Fig 3:**
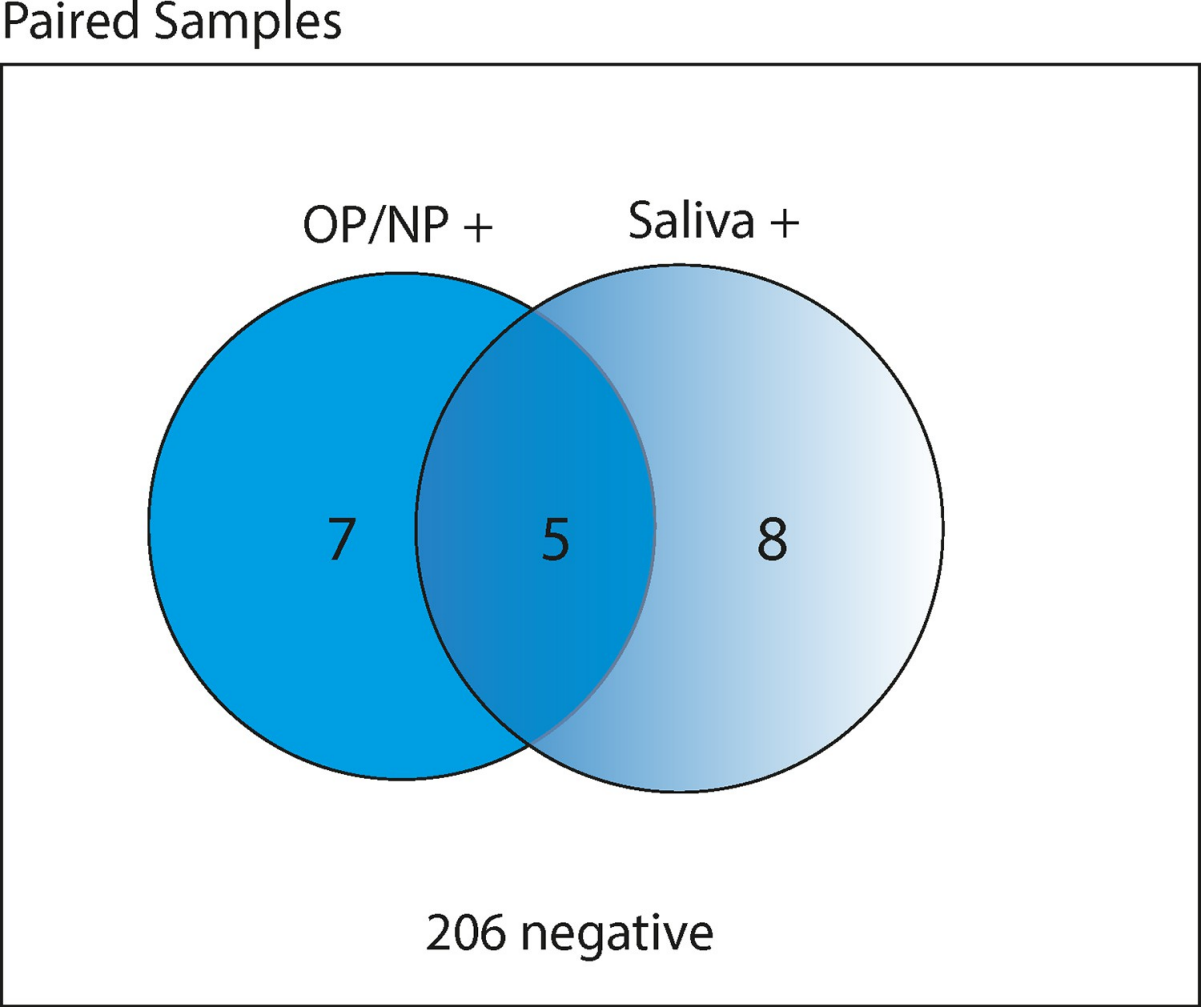
Paired analysis of SARS-CoV-2 RNA in oropharyngeal/nasopharyngeal (OP/NP) and saliva samples. Venn diagram indicating SARS-CoV-2 RNA status of saliva and OP/NP samples that were paired (samples collected by a participant on the same day), by RT-PCR. Numbers indicate pairs of samples.

**Fig 4:**
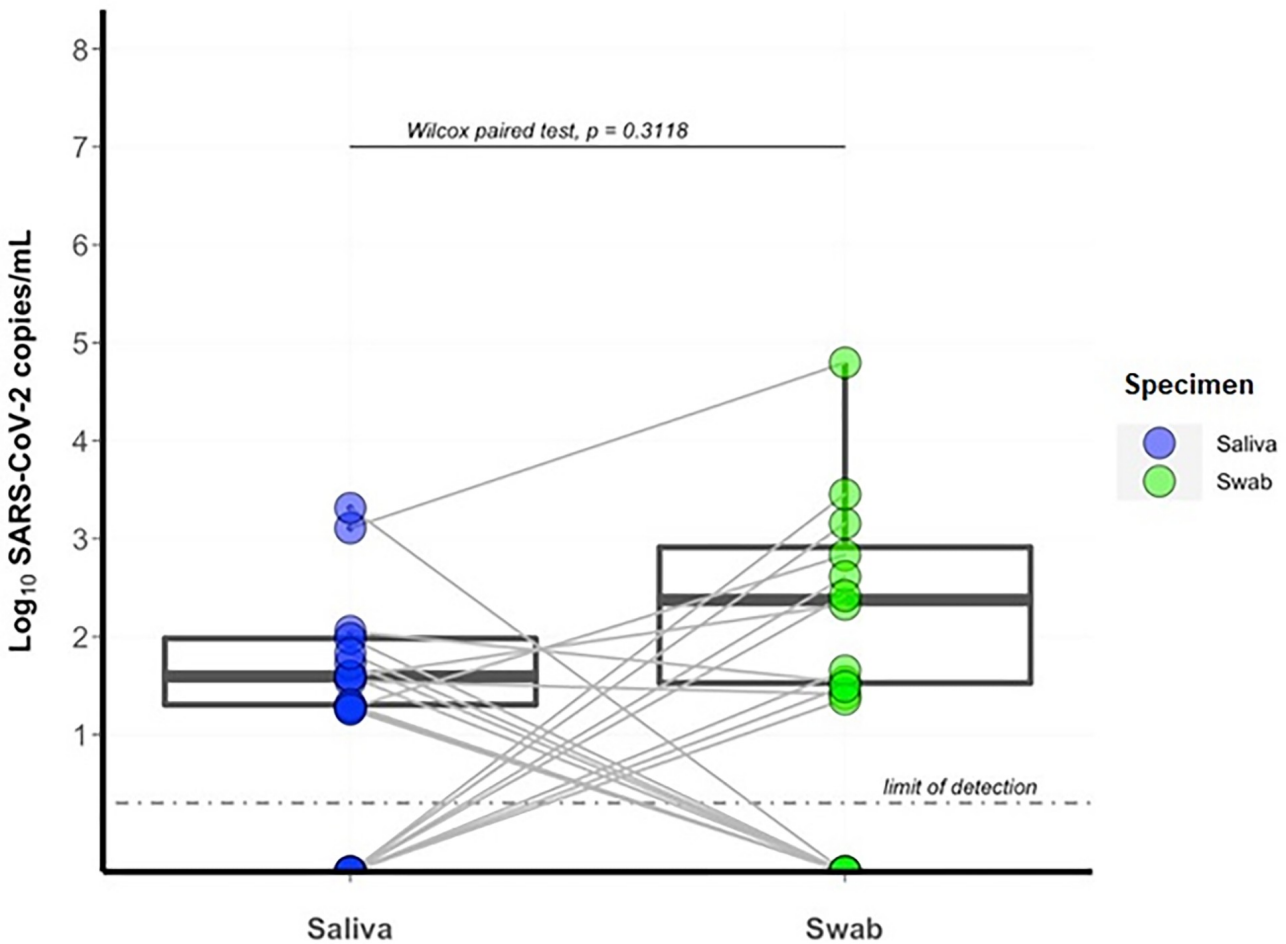
Comparison of SARS-CoV-2 viral load in paired oropharyngeal/nasopharyngeal swab and saliva samples. The logged viral load of paired saliva and nasopharyngeal (NP) samples (n = 20) from healthcare workers for the first 8 weeks of the study period is shown. Paired samples were those where the participant self-collected both saliva and OP/NP sample on the same date. Data points for a single sample are represented by a point outlined by a circle and a single line connects paired samples. Boxes represent interquartile range (IQR) with a median central line.

We then investigated the agreement of OP/NP swabs and saliva for SARS-CoV-2 detection, utilizing paired samples where both saliva and OP/NP samples had been self-collected on the same date. A total of 226 paired samples were collected from 79 participants, of which 8·85% (n = 20) paired samples included a SARS-CoV-2 RNA positive result. This comprised 12 positive OP/NP and 13 positive saliva samples (Fig 3). Of these paired samples, 35·0% (n = 7/20) were positive exclusively by OP/NP swab and 40·0% (n = 8/20) exclusively by saliva. In only 25·0% (n = 5/20) paired samples were the OP/NP and saliva result both positive. Overall, there was agreement between results in 93·4% (n = 211/226) pairs. There was no significant difference between viral loads for either sample type (p = 0·312, Fig 4).

## Discussion

In this study, we describe a cohort of frontline HCW and present results of surveillance OP/NP and saliva samples for SARS-CoV-2 infection during the first peak of the COVID-19 pandemic. A high proportion (40·0%) of frontline healthcare workers had evidence of SARS-CoV-2 infection during study follow up, but the majority (55·9%) of these infections were asymptomatic. This is likely to be a conservative estimate. We classified an infection as symptomatic if symptoms occurred within a fourteen day interval either side of a positive SARS-CoV-2 result. However, the mean incubation period of wild-type SARS-CoV-2 was found to be 6.65 days (95% CI 6.31–6.99) in a recent systematic review and meta-analysis [7].

HCW did not consistently self-isolate and seek testing when respiratory symptoms were experienced. Most infections occurred early in the follow up period, likely reflecting transmission prior to community interventions and widespread use of appropriate transmission-based precautions according to national guidelines. We report low rates of positive concordance of saliva and OP/NP samples for the detection of SARS-CoV-2 by RT-qPCR, with SARS-CoV-2 dual-positivity only found in 25·0% of positive matched sample pairs. In our paired analysis of participant positive samples, 40·0% SARS-CoV-2 infections were not detected by saliva analysis, compared to 35·0% missed by OP/NP swab. However, from spiked saliva samples, SARS-CoV-2 was detected with greater sensitivity in saliva compared to OP/NP swabs, by 100-fold. This study supports the use of asymptomatic screening in healthcare workers and other highly exposed individuals and suggests that testing of weekly saliva, in addition to regular OP/NP sampling, would improve SARS-CoV-2 detection in surveillance screening programmes.

### Strengths and weakness of the study and relation to previous findings

This is a unique cohort study, of highly exposed healthcare workers during a period of intense SARS CoV-2 transmission, prior to routine asymptomatic screening, including both regular OP/NP and saliva sampling. As routine asymptomatic staff testing was not available during the study period and laboratory capacity at the time did not permit contemporaneous analysis, the study results were not available until after the study period. Therefore, participant symptom reporting and self-isolation behavior was not influenced by the surveillance results. A minority of symptomatic participants with SARS-CoV-2 infection met the COVID-19 case definition. Our finding that 55·9% SARS-CoV-2 infections detected were asymptomatic is considerably higher than predicted by a systematic review and meta-analysis, which reported that the minority of infections were asymptomatic (estimated 20%, 95% CI 17–25) [8]. This supports routine surveillance of asymptomatic individuals during periods of high transmission by testing rather than symptom screening, as an infection prevention and control measure.

Most data pertaining to the performance of saliva for SARS-CoV-2 diagnosis is derived from symptomatic individuals, however, prior to this study, performance in asymptomatic and minimally symptomatic individuals was lacking. A number of studies have concluded relatively good performance of saliva compared to NP and/or OP swab RT-qPCR testing in symptomatic individuals [2,3,5,8–15]. A meta-analysis of 37 studies, the majority of participants of which were symptomatic, found a small difference between the sensitivity of saliva and NP swabs (-3·4% (95% CI -9·9 to 2·1%) in favour of NP swabs, when using a reference standard of positivity by either sample [5]. However, another meta-analysis reported relatively low rates of dual positivity (79%, 95% CI 71 to 86%) of NP swabs and saliva, compared to positivity by either specimen type alone, albeit better than our finding in asymptomatic and minimally symptomatic individuals. By combining oropharyngeal and nasopharyngeal swabs, we likely increased the sensitivity of this sample over a single nasopharyngeal or oropharyngeal swab, therefore providing a robust “gold standard” against which to compare the performance of saliva. In our comparison of sample types, we cannot rule out some irregularity of self-sampling technique as participants were not supervised. However, in many settings self-sampling is used for surveillance testing and therefore our findings are generalizable to a real world setting. We do not expect SARS-CoV-2 RNA yield to have been affected adversely by sample transport or storage conditions, which were standardized to minimize variability [16,17]. Symptoms may have been under-reported. However as symptom questionnaires were contemporaneous to sample collection rather than retrospective, we think this is unlikely.

Our study is limited by some loss to follow up and withdrawal of participants, with resulting missing data. However, the overall retention in the study was good (over 80% at week 8) and overall return of samples was high. The high frequency of sample collection (twice weekly for OP/NP sample and once weekly for saliva) is a strength, as it reduces the likelihood that infections were missed between sampling times. However, participants did not submit OP/NP during periods of self-isolation (motivated by participant symptoms or contact) and infections may have been missed during these periods.

### Implications of this study in current context

Performance of RT-PCR tests for detection of SARS-CoV-2 infection varies by sample and population type (symptomatic vs asymptomatic). OP/NP samples are widely considered gold standard for diagnosis, however saliva may perform better in high risk, asymptomatic populations, detecting infections that may be missed by OP/NP swab sampling. Our findings suggest the highest sensitivity strategy for surveillance is likely to be achieved by combining saliva and OP/NP sample testing. This approach may be recommended in populations where there is a strong case for interrupting transmission, such as healthcare settings and in the context of transmission of variants of concern.

Our finding that HCW did not consistently self-isolate and seek testing when respiratory symptoms were experienced will be of interest to those seeking to understand nosocomial infection risk and behaviour. Resource limitations reducing access to routine diagnostic testing possibly impacted on staff isolation and test-seeking behaviour in the first wave of the UK COVID-19 pandemic. Occupational testing first became available to symptomatic HCW at Liverpool University Hospitals NHS Foundation Trust on 2^nd^ April 2020, shortly after the study commenced but only three participants received a Covid-19 diagnosis through this symptomatic testing. Presenteeism in HCW may be a contributory factor, even in the context of a global respiratory pandemic [18]. Clarification on and reinforcement of self-isolation and testing policies for HCW when symptomatic is advisable, especially given symptomatic infections are likely more transmissible [8]. It is not known whether symptomatic healthcare workers attending work would be more or less likely to adhere to protective behaviours, to prevent onward transmission, such as wearing face masks. Our results provide novel data in support of the policy of implementing asymptomatic screening in UK healthcare settings, especially in the context of high rates of community transmission and easing of government restrictions. Future studies to improve understanding of transmissibility of asymptomatic infection, in the context of HCW using transmission-based precautions, would be valuable to inform the risk-benefit debate around HCW surveillance interventions, which impact on maintenance of safe staffing levels in healthcare settings [18,19].

In summary, we report high rates of SARS-CoV-2 infection in asymptomatic and symptomatic HCW attending work during the first wave of the COVID-19 pandemic in UK, as detected by saliva and OP/NP surveillance sampling, with limited agreement between the two sampling methods. Detection of SARS-CoV-2 by surveillance sampling far exceeded case detection by routine diagnostic testing (targeted to those with symptoms) and the majority of infections were asymptomatic. This study supports the use of saliva as an additional sample for screening for SARS-CoV-2 infection, in order to optimize case detection of symptomatic and asymptomatic infections. Saliva should therefore be considered in addition to OP/NP sample testing by RT-qPCR for SARS-CoV-2 detection, in settings where a high sensitivity screening strategy is required.

## Supporting information

S1 Table Ct values and corresponding SARS-CoV-2 viral load for Oropharyngeal/nasopharyngeal swabs. (DOCX)

S2 Table Ct values and corresponding SARS-CoV-2 viral loads for saliva. (DOCX)

S1 Appendix Participant instructions for home collection of samples. (PDF)
