# Supplementary material for: Detection of SARS-CoV-2 infection by saliva and nasopharyngeal sampling in frontline healthcare workers: An observational cohort study
Source: PLoS One. 2023 Jan 27;18(1):e0280908. doi: 10.1371/journal.pone.0280908 (PMC9882898; doi:10.1371/journal.pone.0280908)
Supplement: S1 Table — (DOCX) [file pone.0280908.s001.docx]

Supplement to: Detection of SARS-CoV-2 infection by saliva and nasopharyngeal sampling in frontline healthcare workers: an observational cohort study.

**Supplementary Table S1 Ct values and corresponding SARS-CoV-2 viral load for oropharyngeal/nasopharyngeal swabs**

| Sample type | Ct value | Viral load |
| --- | --- | --- |
| OP/NP Swab | 37.64 | 7.74E+01 |
| OP/NP Swab | 38.62 | 3.93E+01 |
| OP/NP Swab | 36.79 | 1.39E+02 |
| OP/NP Swab | 39.17 | 2.69E+01 |
| OP/NP Swab | 37.91 | 6.44E+01 |
| OP/NP Swab | 33.43 | 1.43E+03 |
| OP/NP Swab | 38.51 | 2.38E+01 |
| OP/NP Swab | 27.98 | 6.26E+04 |
| OP/NP Swab | 35.20 | 5.52E+02 |
| OP/NP Swab | 38.86 | 3.46E+01 |
| OP/NP Swab | 38.43 | 4.65E+01 |
| OP/NP Swab | 39.10 | 2.59E+01 |
| OP/NP Swab | 39.00 | 3.03E+01 |
| OP/NP Swab | 39.23 | 3.17E+01 |
| OP/NP Swab | 35.42 | 3.61E+02 |
| OP/NP Swab | 38.18 | 2.98E+01 |
| OP/NP Swab | 38.40 | 4.50E+01 |
| OP/NP Swab | 19.51 | 1.78E+07 |
| OP/NP Swab | 38.83 | 3.52E+01 |
| OP/NP Swab | 36.94 | 1.16E+02 |
| OP/NP Swab | 32.95 | 2.00E+03 |
| OP/NP Swab | 40.32 | 1.21E+01 |
| OP/NP Swab | 37.95 | 6.27E+01 |
| OP/NP Swab | 31.03 | 7.59E+03 |
| OP/NP Swab | 39.27 | 2.30E+01 |
| OP/NP Swab | 38.06 | 5.79E+01 |
| OP/NP Swab | 40.37 | 1.22E+01 |
| OP/NP Swab | 33.12 | 1.42E+03 |
| OP/NP Swab | 36.06 | 2.13E+02 |
| OP/NP Swab | 24.06 | 9.47E+05 |
| OP/NP Swab | 35.23 | 4.11E+02 |
| OP/NP Swab | 29.88 | 2.04E+04 |
| OP/NP Swab | 40.18 | 1.74E+01 |
| OP/NP Swab | 39.26 | 3.10E+01 |
| OP/NP Swab | 32.45 | 2.84E+03 |
| OP/NP Swab | 36.20 | 2.62E+02 |
| OP/NP Swab | 34.51 | 6.79E+02 |
| OP/NP Swab | 30.08 | 1.91E+04 |
| OP/NP Swab | 39.56 | 2.68E+01 |
| OP/NP Swab | 29.54 | 1.95E+04 |
| OP/NP Swab | 24.95 | 6.28E+05 |
| OP/NP Swab | 23.65 | 1.55E+06 |
| OP/NP Swab | 22.49 | Not done |
| Median (IQR) | 36.94 (32.45-38.86) | 96.70 (30.83-2210) |
