# Supplementary material for: Detection of SARS-CoV-2 infection by saliva and nasopharyngeal sampling in frontline healthcare workers: An observational cohort study
Source: PLoS One. 2023 Jan 27;18(1):e0280908. doi: 10.1371/journal.pone.0280908 (PMC9882898; doi:10.1371/journal.pone.0280908)
Supplement: S2 Table — (DOCX) [file pone.0280908.s002.docx]

**Supplementary Table S2 Ct values and corresponding SARS-CoV-2 viral loads for saliva**

| Sample type | Ct value | Viral load |
| --- | --- | --- |
| Saliva | 39.45 | 1.92E+01 |
| Saliva | 33.06 | 1.28E+03 |
| Saliva | 37.17 | 1.14E+02 |
| Saliva | 38.72 | 3.53E+01 |
| Saliva | 39.04 | 3.29E+01 |
| Saliva | 38.34 | 5.33E+01 |
| Saliva | 39.51 | 1.84E+01 |
| Saliva | 36.69 | 1.67E+02 |
| Saliva | 37.50 | 9.59E+01 |
| Saliva | 39.24 | 2.43E+01 |
| Saliva | 39.59 | 1.75E+01 |
| Saliva | 38.78 | 3.38E+01 |
| Saliva | 38.01 | 6.72E+01 |
| Saliva | 40.10 | 2.02E+01 |
| Saliva | 28.59 | 2.83E+04 |
| Saliva | 32.70 | 2.06E+03 |
| Saliva | 37.13 | 1.59E+02 |
| Saliva | 38.89 | 3.46E+01 |
| Saliva | 39.15 | 1.87E+01 |
| Saliva | 39.83 | 1.80E+01 |
| Saliva | 40.76 | 1.06E+01 |
| Saliva | 26.60 | 1.41E+05 |
| Median (IQR) | 38.75 (37.02-39.47) | 34.95 (19.08-161.0) |
