# Supplementary material for: Detection of SARS-CoV-2 infection by saliva and nasopharyngeal sampling in frontline healthcare workers: An observational cohort study
Source: PLoS One. 2023 Jan 27;18(1):e0280908. doi: 10.1371/journal.pone.0280908 (PMC9882898; doi:10.1371/journal.pone.0280908)
Supplement: S1 Appendix — (PDF) [file pone.0280908.s003.pdf]

Supplement to: Detection of SARS-CoV-2 infection by saliva and nasopharyngeal sampling in frontline healthcare workers: an observational cohort study.

Appendix: Instructions for Participants on self-sampling

	**Bloods**	**Nasal and throat swabs**	**Nasal strip**	**Saliva**
**When?**	At enrolment and then every four weeks	Twice a week	Once a week	
**How?**	Research nurse will take sample at your workplace	Self-sampling at home	Self-sampling at home	
**Collection**	Research team will transport sample back to lab	Bring the sample to work on day of collection; drop in sample collection box in ward area	Store sample in the storage container in your home freezer. Research team will arrange collection (usually once per month when meeting for blood sample). Samples needs to be in the laboratory within 2 hours of removal from freezer.	

Before and after taking your samples: wash your hands for 20 seconds with soap & warm water.

Nasal and Throat Virology Swabs: Please collect this twice per week prior to going in to work. Please do the swab yourself rather than asking a friend, family member or colleague to take the swab to minimise possible transmission.

1) Swab both tonsillar arches on both sides by swiping twice on each side.
2) Put swab into the fluid in the collection tube.
3) Use the plain swab then place into your nostril advancing (as per image) and twist a minimum of two times.
4) Return the swab into the same sample collection fluid.
5) Break the swab handle at the moulded break point
6) Close the lid tightly.
7) Ensure the sample is labelled correctly.
8) Place in a sample bag.
9) Complete the symptom questionnaire and place in bag with sample.
10) Complete the time and date on the sample table.
11) Place bagged sample in the collection box on your ward on the same day as collected.

Nasal Swab

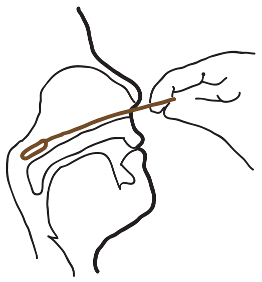

Saliva Samples - Please collect this sample once per week.
Please avoid drinking, eating, chewing gum or brushing your teeth for 30 minutes before giving the saliva sample. We recommend doing this as soon as you get up.

It is important that you are not coughing up phlegm from your throat but produce clear/white "spit" from the mouth. If the saliva is frothy or there are a lot of bubbles, please add more saliva.

1) Screw (not push) the saliva funnel to the top of the saliva tube (a).
2) Spit into the funnel then allow the saliva to run down the tube to at least the fill line.
3) Change the funnel top (b) to the white screw top (c) then shake the tube.
4) Place in a sample bag.
5) Store in the Tupperware storage container in your freezer at home.
6) Record sample dates on sample

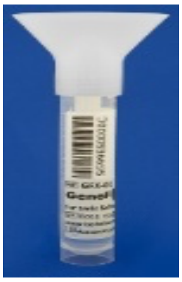
a) Saliva tube

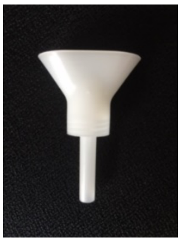
b) funnel top

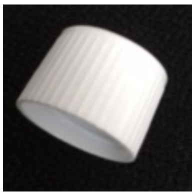
c) white screw top

NB: The saliva sample pot has liquid in it to preserve the sample. The ingredients are classified as "not a hazardous substance or mixture". This should still not be swallowed. In case of any accidental spillage, this can be cleaned with a kitchen towels and the surface washed with soapy water. Do not try to put escaped liquid back in the tube.

Nasal Strip sample - Please collect this sample once per week.

1) Unscrew the strip from the container
2) Set a timer on your phone for 2 minutes
3) Place the strip in one nostril touching the inside of your nose and keeping it in place by pressing gently on the nostril for two minutes.
4) Return strip to container.
5) Ensure sample is labelled correctly.
6) Place into Tupperware storage container in freezer.

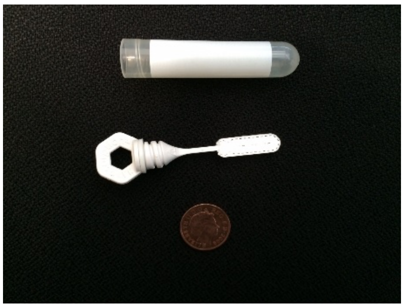

After taking your samples: wash your hands for 20 seconds with soap & warm water.
Record sample dates on personalised sample schedule.
